# Temperature-responsive regulation of the polycyclic aromatic hydrocarbon-degrading mesophilic bacterium *Novosphingobium pentaromativorans* US6-1 with a temperature adaptation system

**DOI:** 10.1128/aem.01484-24

**Published:** 2024-12-12

**Authors:** Zhuangzhuang Liu, Xinran Liu, Haiyan Huang, Feifei Cao, Qiu Meng, Tingheng Zhu, Jianhua Yin, Xiaofei Song, Zhiliang Yu

**Affiliations:** 1 College of Biotechnology and Bioengineering, Zhejiang University of Technology 630365 , Hangzhou, Zhejiang, China; 2 Hangzhou Chuhuan Science and Technology Co. Ltd., Hangzhou, Zhejiang, China; Unversidad de los Andes, Bogotá, Colombia

**Keywords:** temperature-responsive system, synthetic biology, polycyclic aromatic hydrocarbons, biodegradation, *Novosphingobium pentaromativorans*

## Abstract

Survivability and tolerance of polycyclic aromatic hydrocarbon (PAH)-degrading bacteria in harsh environments, especially under varying temperatures, are a bottleneck for the effective application of *in situ* bioremediation. In this study, a temperature adaptation system (TAS) was constructed by combining a customized thermotolerant system with a customized cold-resistant system to realize the temperature-responsive regulation of the PAH-degrading mesophilic bacterium *Novosphingobium pentaromativoran*s US6-1. The innovative dual-pronged TAS strategy enabled the chassis strain to effectively tackle conditions under varying temperatures, ensuring robust biological activities across a broadened temperature spectrum and exhibiting the potential to realize the high-efficiency PAH degradation of *N. pentaromativorans* US6-1 in *in situ* bioremediation. Furthermore, the temperature-responsive regulation achieved using the TAS circuit is likely promising for creating intelligent microbial cell factories and avoiding precise temperature maintenance, making it highly useful for industrial applications.

**IMPORTANCE:**

Environmental temperature is among the extremely important factors that determine the bioactivities of pollutant-degrading microorganisms in *in situ* bioremediation. Effectively maintaining the survivability and tolerance of mesophilic microorganisms under harsh conditions and varying temperatures remains a challenge in the application of pollutant bioremediation. This study, for the first time, developed a temperature adaptation system by combining a customized thermotolerant system with a customized cold-resistant system to realize the temperature-responsive regulation of the polycyclic aromatic hydrocarbon (PAH)-degrading mesophilic bacterium *Novosphingobium pentaromativoran*s US6-1, thus diminishing the need for precise temperature control in PAH bioremediation.

## INTRODUCTION

Polycyclic aromatic hydrocarbons (PAHs) are aromatic compounds composed of two or more fused benzene rings that are primarily generated through the incomplete combustion or pyrolysis of organic substances (1). PAHs are pervasive pollutants found across a variety of environments, where they cause harmful biological impacts such as toxicity, mutagenicity, and carcinogenicity (2). PAHs in the environment can be degraded through biological, physical, and chemical processes. Among these methods, microbial degradation has drawn significant attention for its effectiveness, eco-friendliness, cost-effectiveness, and sustainability (3). In previous work, *Novosphingobium pentaromativorans* US6-1, a mesophilic marine bacterium with an optimal growth temperature of 30°C, was isolated from the muddy sediment of Ulsan Bay, South Korea. Research has shown that *N. pentaromativorans* US6-1 has the ability to degrade chrysene, phenanthrene, pyrene, benzo[*b*]fluoranthene, benz[*a*]anthracene, and benzo[*a*]pyrene (4). Due to its diverse metabolic abilities, this bacterium is considered a promising candidate for the bioremediation of PAH-contaminated areas (4).

Bioremediation efforts can typically be categorized as either *ex situ* or *in situ* bioremediation based on their location (5). *Ex situ* remediation involves removing contaminated soil or groundwater from their original location and transporting them to a different location for cleanup. By contrast, *in situ* techniques directly address pollutants on-site and are often considered the most cost-effective and environmentally friendly treatment approach (6). PAHs are distributed worldwide, almost at all latitudes and in low- to high-altitude areas, due to long-term anthropogenic pollution activities (7). Considering the energy consumption and degradation efficiency of microbes, it is unfeasible and unrealistic to provide ideal conditions for microbial growth and metabolism during *in situ* remediation (8). Providing the appropriate temperature during *in situ* remediation is particularly challenging. However, accomplishing microbial degradation is difficult under inappropriate temperatures because extreme temperatures negatively affect cell growth and division, damage plasma membrane structure and function, and inhibit protein synthesis (9). Super-robust PAH-degrading microorganisms capable of adapting to a wider temperature range in different environments are thus highly desirable.

Synthetic biology aims to systematize the design of genetically encoded biological systems based on the design-build-test-learn (DBTL) engineering principles (10). Consequently, synthetic biology can provide effective methods to incorporate advanced biological functions within and between cells and improve the characteristics of current microbial biotransformation systems by equipping cells with tools and elements to enhance stress resistance (11). For example, Jia et al. developed a gene network called the intelligent microbial heat-regulating engine (IMHeRE) to enhance the thermorobustness of *Escherichia coli* by incorporating a thermotolerant system and a quorum-regulating system (12). Li et al. designed an advanced genetic circuit known as genetic pH shooting (GPS) to enable microbes to self-regulate their pH levels (13). Alper et al. created a tool called global transcription machinery engineering (gTME) and enhanced *E. coli* tolerance to high ethanol stress and various other chemicals, including acetate, hexane, and *p*-hydroxybenzoic acid (14). Throughout the process of biological evolution, extremophiles have evolved distinctive coping strategies to address variations in environmental factors. These strategies may hold potential as novel tools for synthetic biology to enhance microbial robustness under the strict conditions necessary for different industrial applications. Researchers have investigated the use of a single key protein or larger natural protein complexes from extremophiles to develop promising hosts with enhanced tolerance to various stressors, such as temperature, solvent concentration, acidity, and oxidative stress (15–17), demonstrating the potential applications of these natural mechanisms in engineering robustness in microbial hosts.

**Fig 1:**
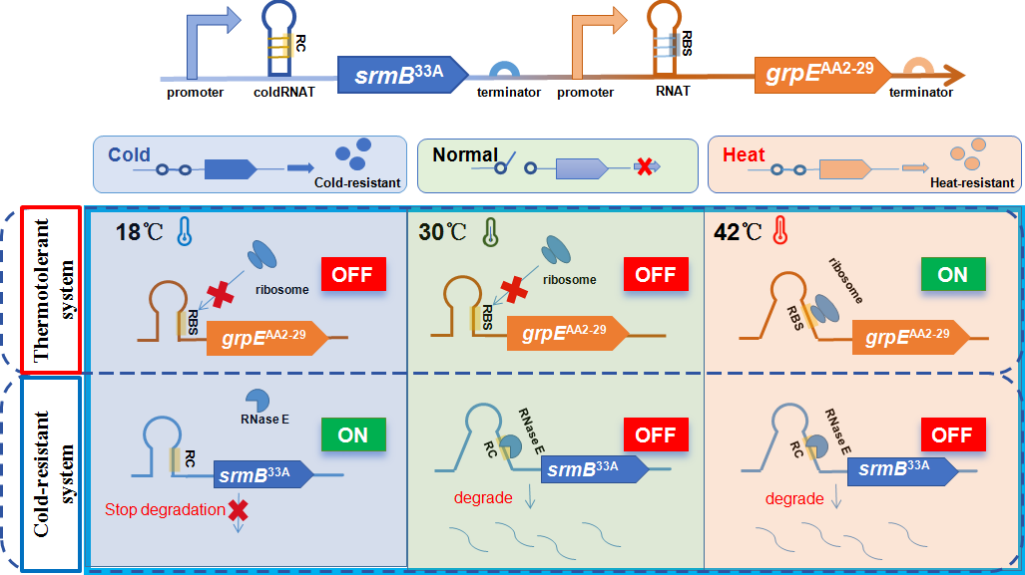
Mechanism of temperature adaptation system (TAS). The TAS circuit was constructed by merging a customized thermotolerant system with a customized cold-resistant system. In the thermotolerant system, when the temperature is normal (30°C) or low (18°C), the heat-induced RNA thermometers (heat-RNATs) form a hairpin structure to prevent ribosomes from binding to mRNA and inhibit the expression of heat shock proteins (HSPs), and the thermotolerant system is in the “OFF” state. When the temperature is high (42°C), the hairpin structure of heat-RNATs is opened, allowing the ribosomes to bind to mRNA and express HSPs, and the system is in the “ON” state. In the cold-resistant system, at normal (30°C) or high temperatures (42°C), the hairpin structure of cold-induced RNA thermosensors (cold-RNATs) is opened to expose the RNase E cleavage site (RC), allowing RNase E to degrade mRNA, inhibiting the expression of cold-induced proteins (CIPs), thereby keeping the cold-resistant system in the “OFF” state. At low temperatures (18°C), the cold-RNATs form a hairpin structure, preventing RNase E from degrading mRNA and expressing CIPs, and the system is in the “ON” state.

In this study, drawing inspiration from extremophiles adapted to adverse temperature environments, a temperature-responsive adaptation system (TAS) was constructed using synthetic biology to realize the temperature-responsive regulation of the PAH-degrading bacterium *N. pentaromativorans* US6-1 (Fig. 1). The developed TAS circuit was assembled by combining a customized thermotolerant system with a customized cold-resistant system. The customized thermotolerant system was constructed with heat shock proteins (HSPs) and heat-induced RNA thermometers (heat-RNATs), of which HSPs play a pivotal role in safeguarding cellular integrity against thermal insults, whereas heat-RNATs act as gene expression regulators to mediate the expression of HSPs in response to heat shock. Similarly, the customized cold-resistant system was constructed by merging cold-induced proteins (CIPs) with cold-induced RNA thermosensors (cold-RNATs). This innovative dual-pronged TAS strategy allowed *N. pentaromativorans* US6-1 to effectively tackle varying temperatures, ensuring its robust biological activity and high-efficiency PAH degradation under conditions with a broadened temperature range from 18°C to 42°C.

## RESULTS

### Customized thermotolerant system for the PAH-degrading bacterium *N. pentaromativorans* US6-1

**Fig 2:**
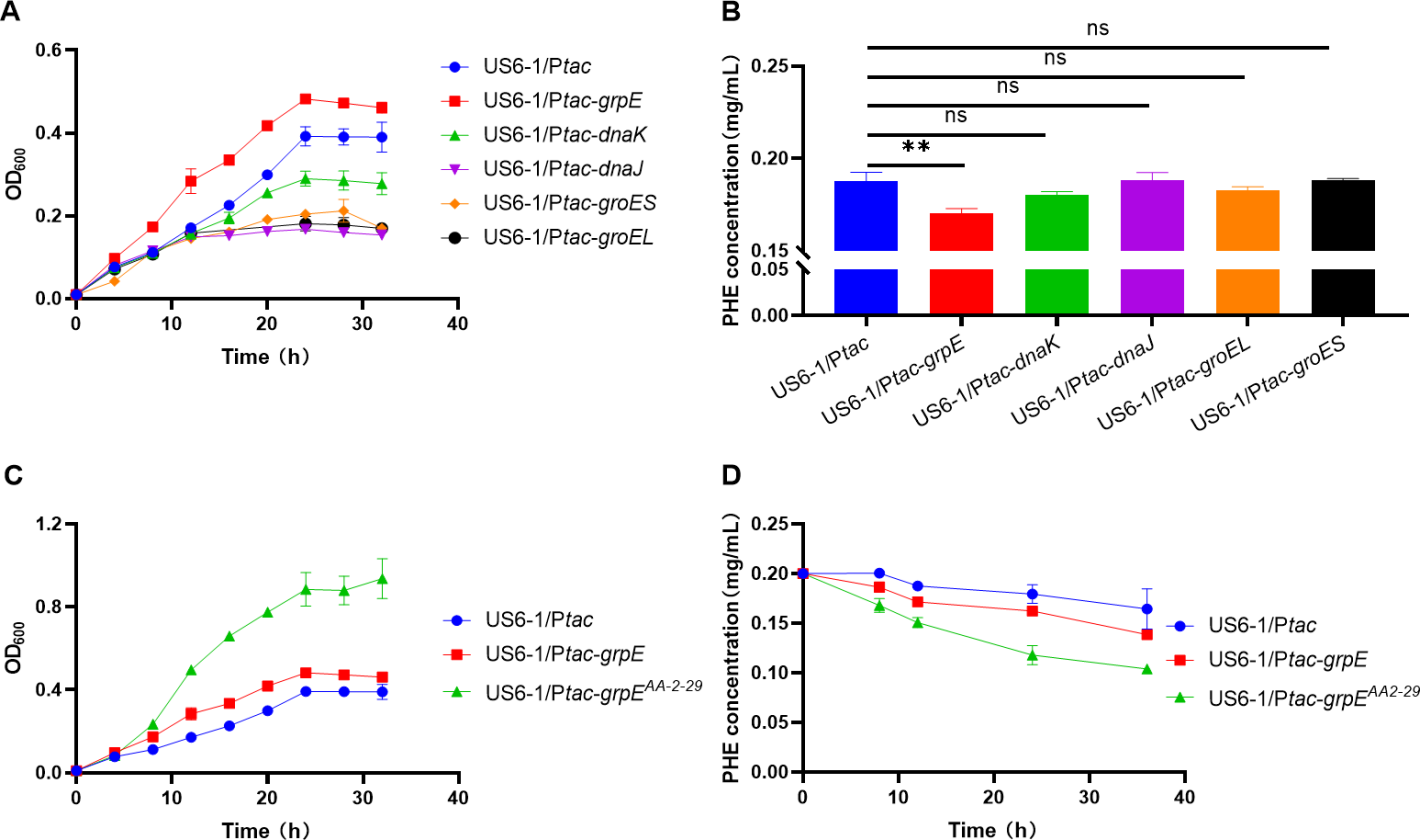
Effect of heat-resistant elements on the heat resistance of chassis strains at 42°C. (**A**) Growth curve of endogenous heat shock protein overexpressed strains. Cells were cultured in a P5Y3 medium. (**B**) Residual phenanthrene of endogenous heat shock protein overexpresssed strains after degradation for 24 h. Cells were cultured in a P5Y3 medium with 0.2 mg/mL phenanthrene. (**C**) Effect of the expression of endogenous *grpE* and exogenous *grpE* from *Thermus thermophilus* AA2-29 (*grpE*^AA2-29^) on the growth of *N. pentaromativorans* US6-1. Cells were cultured in a P5Y3 medium. (**D**) Effects of the expression of endogenous *grpE* and exogenous *grpE*^AA2-29^ on phenanthrene degradation of *N. pentaromativorans* US6-1. Cells were cultured in a P5Y3 medium with 0.2 mg/mL phenanthrene. US6-1/P*tac*: wild-type strain of *N. pentaromativorans* US6-1 containing blank plasmid vector P*tac*; US6-1/P*tac-grpE: N. pentaromativorans* US6-1 with the overexpressed endogenous *grpE* under control of the *tac* promoter; US6-1/P*tac-dnaK: N. pentaromativorans* US6-1 with the overexpressed endogenous *dnaK* under control of the *tac* promoter; US6-1/P*tac-danJ: N. pentaromativorans* US6-1 with the overexpressed endogenous *danJ* under control of the *tac* promoter; US6-1/P*tac-groEL: N. pentaromativorans* US6-1 with the overexpressed endogenous *groEL* under control of the *tac* promoter; US6-1/P*tac-groES: N. pentaromativorans* US6-1 with the overexpressed endogenous *groES* under control of the *tac* promoter; and US6-1/P*tac-grpE*^AA2-29^: *N. pentaromativorans* US6-1 with the heterologously expressed *grpE*^AA2-29^ under control of the *tac* promoter. PHE: phenanthrene. Unless otherwise specified, the expression of genes under the control of the *tac* promoter was induced by 0.1 mM isopropyl *β*-D-1-thiogalactopyranoside (IPTG). Significant difference: ns, *P* > 0.05; **, *P* < 0.01.

Cells are equipped with an intricate protein quality control network that reacts swiftly to heat shock by expressing a diverse array of molecular chaperones and proteases (18). This vital mechanism effectively addresses heat-induced protein denaturation either by maintaining protein conformation or by degrading compromised proteins, ultimately reinstating cellular homeostasis (19). In this study, detecting the transcription levels of HSPs as the temperature increased revealed that the expression levels of endogenous HSPs (Table S1) in *N. pentaromativorans* US6-1 were positively associated with high temperatures at the transcription level (Fig. S1), indicating that these endogenous HSPs may participate in heat shock adaption. Further experiments verified that the overexpression of endogenous HSPs in *N. pentaromativorans* US6-1 enhanced cellular heat tolerance. (note: cell growth is the direct indication of cell stress resistance or tolerance. Thus, here, the growth curves were used to reflect the tolerance or resistance to harsh temperatures.) More specifically, the OD_600_ of strains with the overexpression of *grpE* and *dnaK*, two HSP genes, outperformed the OD_600_ of the control strain (US6-1/P*tac*) by approximately 1.2 times when incubated at 37°C for 24 h (Fig. S2A). In addition, both overexpression strains exhibited superior abilities to degrade phenanthrene, a type of PAHs. Notably, the *grpE* overexpression strain (US6-1/P*tac-grpE*) exhibited significantly reduced residual phenanthrene of only 26.0% at 8 h relative to the control strain (US6-1/P*tac*) (Fig. S2B). There is no doubt that the overexpression of heterologous proteins may result in a metabolic burden on the cells (20). Even so, at 42°C, the *grpE* overexpression strain exhibited enhanced growth and phenanthrene degradation abilities (Fig. 2A and B), with OD_600_ of 1.23 times that of the control strain and residual phenanthrene of 90.7% that of the control strain at 24 h. In addition, cell morphology analysis revealed that the *grpE* overexpression cells maintained normal morphology at 42°C, whereas the control cells did not (Fig. S3), further indicating that GrpE plays an extraordinarily crucial role in safeguarding *N. pentaromativorans* US6-1 cells from damage caused by high temperature.

Although the overexpression of endogenous HSPs indeed enhanced the heat resistance of the chassis cells to a certain degree, this protective effect may be inadequate at higher temperatures. In addition, the protective capability of intrinsic HSPs to the cells is transient and somewhat restricted (21). Exploring a more extensive and efficient array of HSPs is a promising approach to address this challenge. Thermophilic bacteria represent a plentiful natural reservoir of HSPs that may provide abundant thermotolerant elements for a customized thermotolerant system. To guarantee biocompatibility between the exogenous thermotolerant components and the recipient strains, a phylogenetic tree was established based on bioinformatics analyses by comparing the HSPs between both chassis and thermophilic strains (Fig. S4). The results showed that HSPs Dnak/J from *T. thermophilus* AA2-29 exhibited a high degree of homology with their counterparts in *N. pentaromativorans* US6-1. However, the homology of the nucleic acid exchange factor GrpE was notably low at only 22.89%. Considering the endogenous overexpression data above and the reported synergistic roles of GrpE, DnaK, and DnaJ in protein quality control (22, 23), it was hypothesized that the *grpE* gene may be the decisive factor in the high-temperature tolerance of thermophilic bacteria. To verify this hypothesis and further enhance the ability of *N. pentaromativorans* US6-1 to resist high temperatures, the *grpE* gene from *T. thermophilus* AA2-29 (*grpE*^AA2-29^) was commercially synthesized and heterologously expressed in *N. pentaromativorans* US6-1. The results showed that the heterologous expression of *grpE*^AA2-29^ improved the heat resistance of *N. pentaromativorans* US6-1 at 42°C, with OD_600_ of 2.25 times that of the control strain (US6-1/P*tac*) at 24 h, far greater than the increase of 1.23 times achieved by the endogenous overexpression strain (US6-1/P*tac-grpE*) compared with the control strain (US6-1/P*tac*) (Fig. 2C). The phenanthrene degradation at 42°C was also evaluated. The residual phenanthrene of US6-1/P*tac-grpE*^AA2-29^ and US6-1/P*tac-grpE* was 65.9% and 90.5% that of the control strain (US6-1/P*tac*) at 24 h, respectively (Fig. 2D). Collectively, these results demonstrated that exogenous *grpE*^AA2-29^ is superior to the endogenous *grpE* of *N. pentaromativorans* US6-1 in terms of thermotolerance. Therefore, *grpE*^AA2-29^ was selected as an ideal heat-resistant element for the creation of a customized thermotolerant system in *N. pentaromativorans* US6-1. It has been reported that GrpE is a molecular chaperone to help the correct folding of proteins (22). The GrpE derived from thermophilic bacteria may help the correct folding of denatured proteins produced by high temperatures, thus reducing the damage caused by high temperatures to bacteria.

### Control of the thermotolerant system for heat-responsive regulation in *N. pentaromativorans* US6-1

**Fig 3:**
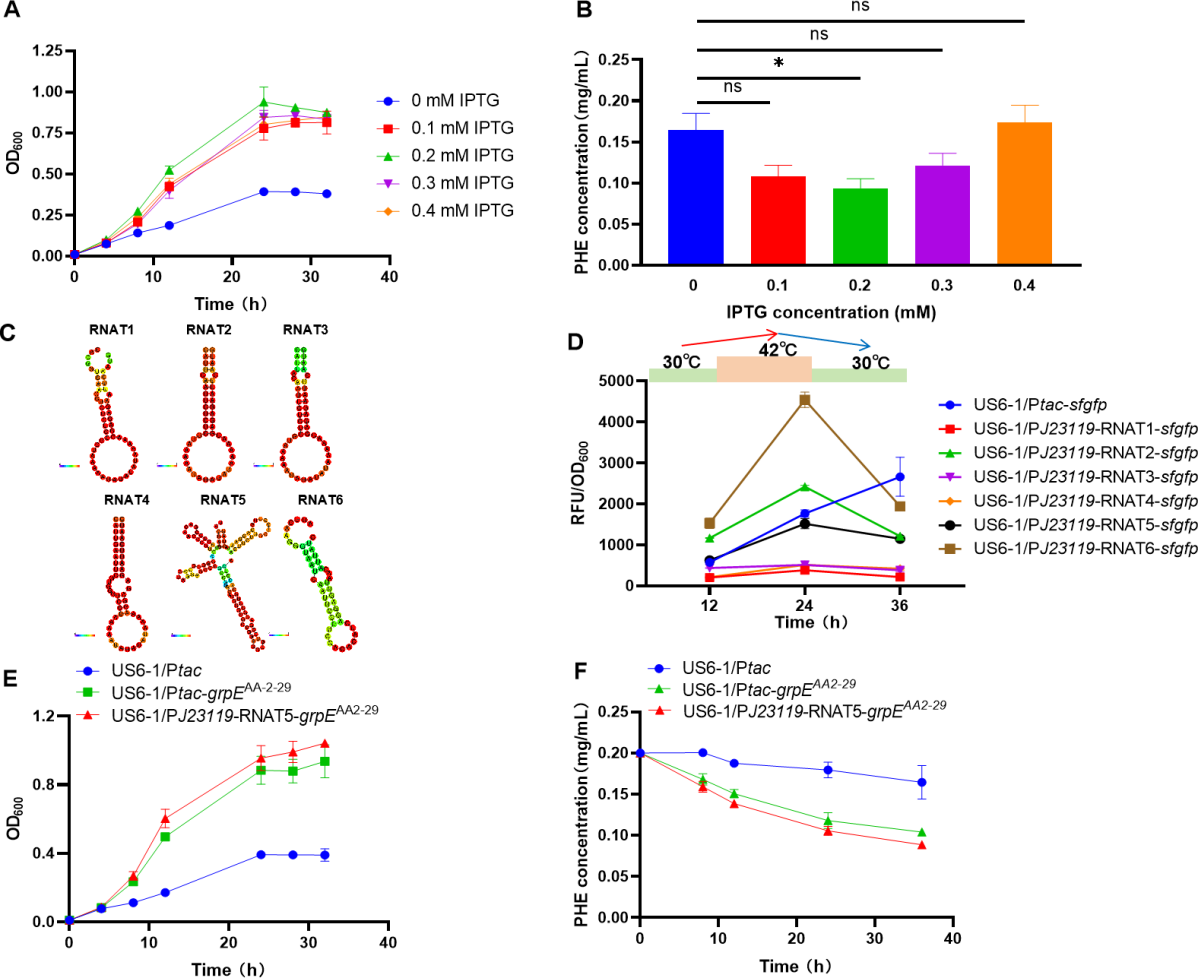
Construction and evaluation of heat-induced genetic switch. (**A and B**) The inducible promoter P*tac* was used to control the expression of heat-resistant element *grpE* from *T. thermophilus* AA2-29 (*grpE*^AA2-29^) in *N. pentaromativorans* US6-1 (US6-1/P*tac-grpE*^AA2-29^) with different IPTG concentrations, and effects of the *grpE*^AA2-29^ expression on the cell growth (**A**) and phenanthrene degradation (**B**) at 42°C for 24 h were evaluated. Cells were cultured in P5Y3 medium for measurement of the growth curve, whereas they were cultured in a P5Y3 medium with 0.2 mg/mL phenanthrene for measurement of the phenanthrene degradation curve. (**C**) Predicted structure of the candidate heat-induced switches (heat-RNATs). (**D**) Evaluation of heat-induced switches constructed by combining heat-RNATs with constitutive promoter P*J23119*. The reporter gene *sfgfp* was used to explore the optimal heat-induced switch for control of the thermotolerant system, and the *tac* promoter was used as the control of chemical IPTG-induced switch for comparison. Cells were first cultured at 30°C for 12 h, then transferred to 42°C (0.2 mM IPTG was immediately added to the control of chemical IPTG-induced switch to induce the expression of *sfgfp*) for 12 h, and finally transferred back to 30°C for another 12 h. US6-1/P*tac-sfgfp: N. pentaromativorans* US6-1 with the heterologously expressed *sfgfp* under control of the *tac* promoter; US6-1/P*J23119*-RNAT1-*sfgfp* to US6-1/P*J23119*-RNAT6-*sfgfp: N. pentaromativorans* US6-1 with the heterologously expressed *sfgfp* under control of constitutive promoter P*J23119* combined with different heat-induced RNATs (RNAT1 to RNAT6). (**E and F**) Effects of optimized heat-induced switch and chemical IPTG-induced switch on the cell growth (**E**) and phenanthrene degradation (**F**) at 42°C. Cells were cultured in P5Y3 medium for measurement of the growth curve, whereas they were cultured in a P5Y3 medium with 0.2 mg/mL phenanthrene for measurement of the phenanthrene degradation curve. US6-1/P*tac*: wild-type strain of *N. pentaromativorans* US6-1 containing blank plasmid vector P*tac*; US6-1/P*tac-grpE*^AA2-29^: *N. pentaromativorans* US6-1 with the heterologously expressed *grpE*^AA2-29^ under control of the *tac* promoter; US6-1/P*J23119*-RNAT5-*grpE*^AA2-29^: *N. pentaromativorans* US6-1 with the heterologously expressed *grpE*^AA2-29^ under control of constitutive promoter P*J23119* combined with heat-induced switch RNAT5. PHE: phenanthrene. Unless otherwise specified, the expression of genes under the control of the *tac* promoter was induced by 0.2 mM IPTG. Significant difference: ns, *P* > 0.05; *, *P* < 0.05.

HSPs are crucial factors in cellular adaption to high temperatures, but the overexpression of HSPs at normal temperatures may result in unnecessary energy consumption, substrate consumption, and detrimental effects on the production process (12). Therefore, modulating the expression of heat-resistant elements is essential to minimize side effects. To determine the optimal expression level of heat-resistant elements at 42°C, different concentrations of isopropyl *β*-D-1-thiogalactopyranoside (IPTG) were utilized to control the activity of the inducible promoter P*tac,* and the expression level of *grpE*^AA2-29^ was accordingly optimized by evaluating cell growth and phenanthrene degradation. Although there was no notable disparity in cell growth under different IPTG concentrations, the strain US6-1/P*tac-grpE*^AA2-29^ exhibited the most optimal growth at 42°C under 0.2 mM IPTG (Fig. 3A). The residual phenanthrene of the strain US6-1/P*tac-grpE*^AA2-29^ at 42°C for 24 h was also the lowest under 0.2 mM IPTG, which is 57.1% of that of the control without IPTG induction (Fig. 3B). These findings indicated that the P*tac* promoter achieved the most appropriate expression intensity of *grpE*^AA2-29^ under 0.2 mM IPTG, providing an ideal “ON” state for a heat-induced switch.

As opposed to using chemical induction with IPTG, which is useful to induce gene expression but is potentially toxic to cells (Fig. 3B), a temperature-responsive regulation system in *N. pentaromativorans* US6-1 is more meaningful for practical applications. Drawing inspiration from the survival mechanisms of bacteria by autonomously altering gene expression in response to different environmental temperatures (24), temperature-sensing RNA sequence-based heat-RNATs have been developed as heat-responsive regulation switches to quickly control the expression of genes by sensing the environmental temperatures (25, 26). At low temperatures, heat-RNATs are situated in the 5’ untranslated region of mRNA and fold into secondary structures to hinder translation by sequestering ribosome binding sites (RBSs). As the temperature increases, heat-RNATs expose the RBSs, enabling the expression of previously blocked genes. To construct a reporter gene cassette in *N. pentaromativorans* US6-1 comprising a constitutive promoter, heat-RNATs, and the green fluorescent protein gene (*sfgfp*, a reporter gene) for assessing the functionality of various heat-induced switches, a constitutive promoter (P*J23119*) was selected from the iGEM Registry. Six heat-RNATs, including four heat-RNATs (RNAT1, RNAT2, RNAT3, and RNAT4) (Table S2), were designed using the UNAFold Web Server (http://www.unafold.org/). In addition, two heat-RNATs (RNAT5 and RNAT6) (Table S3) screened from the existing database were employed, and their structures were predicted using the RNAfold website (http://rna.tbi.univie.ac.at/cgi-bin/RNAWebSuite/RNAfold.cgi) (Fig. 3C). Accordingly, six combinations (six heat-RNATs × one constitutive promoter) were tested to explore optimal heat-induced switches for the control of the thermotolerant system (Fig. 3D). Comparing the relative fluorescence intensity caused by the heat-induced switches with that caused by the P*tac* promoter under 0.2 mM IPTG at 42°C demonstrated the heat-induced switch P*J23119*-RNAT5 had the most ideal “ON” state (Fig. 3D). Afterward, the customized thermotolerant system of P*J23119*-RNAT5 was employed as a heat-induced switch to control the expression of *grpE*^AA2-29^ and introduced into *N. pentaromativorans* US6-1 (US6-1/P*J23119*-RNAT5-*grpE*^AA2-29^). As shown in Fig. 3E and F, the OD_600_ and residual phenanthrene of US6-1/P*J23119*-RNAT5-*grpE*^AA2-29^ at 24 h were 2.43 times and 58.1% that of the control strain (US6-1/P*tac*), respectively. Therefore, the optimized heat-induced switch P*J23119*-RNAT5 has comparable performance to the chemical IPTG-induced switch in regulating the thermotolerance of strain US6-1.

### Customized cold-resistant system for the PAH-degrading bacterium *N. pentaromativorans* US6-1

**Fig 4:**
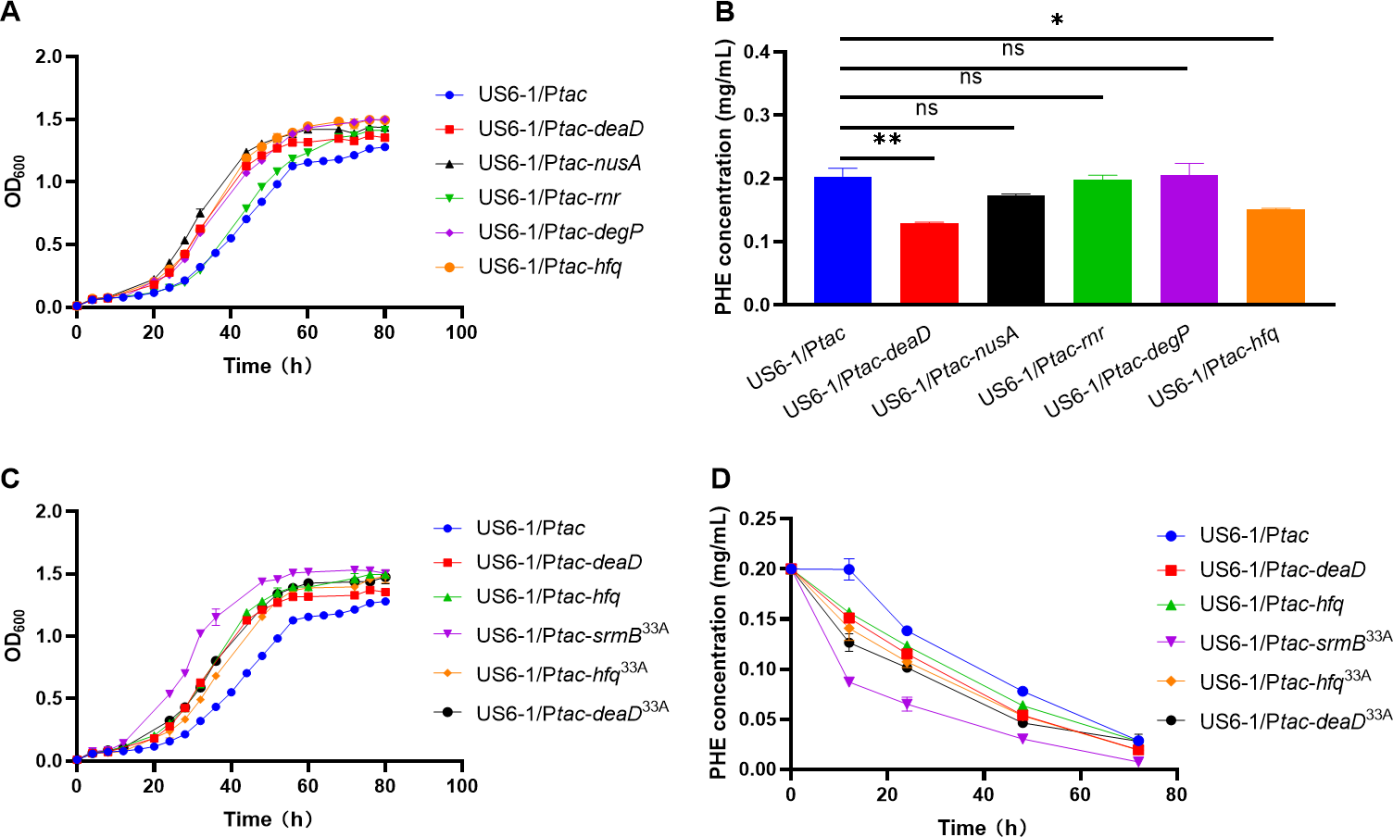
Effect of cold-resistant genetic elements on cold tolerance of chassis strains at 18°C. (**A**) Growth curve of endogenous cold-tolerant element overexpressed strains. Cells were cultured in a P5Y3 medium. (**B**) Residual phenanthrene after degradation for 24 h of endogenous cold-tolerant element overexpressed strains. Cells were cultured in a P5Y3 medium with 0.2 mg/mL phenanthrene. (**C**) Effect of the expression of endogenous cold-tolerant elements and exogenous cold-tolerant elements from *Glaciecola* sp. 33A on the growth of *N. pentaromativorans* US6-1. Cells were cultured in a P5Y3 medium. (**D**) Effect of the expression of endogenous cold-tolerant elements and exogenous cold-tolerant elements from *Glaciecola* sp. 33A on phenanthrene degradation of *N. pentaromativorans* US6-1. Cells were cultured in a P5Y3 medium with 0.2 mg/mL phenanthrene. US6-1/P*tac*: wild-type strain of *N. pentaromativorans* US6-1 containing blank plasmid vector P*tac*; US6-1/P*tac-deaD: N. pentaromativorans* US6-1 with the overexpressed endogenous *deaD* under control of the *tac* promoter; US6-1/P*tac-nusA: N. pentaromativorans* US6-1 with the overexpressed endogenous *nusA* under control of the *tac* promoter; US6-1/P*tac-rnr: N. pentaromativorans* US6-1 with the overexpressed endogenous *rnr* under control of the *tac* promoter; US6-1/P*tac-degP: N. pentaromativorans* US6-1 with the overexpressed endogenous *degP* under control of the *tac* promoter; US6-1/P*tac-hfq: N. pentaromativorans* US6-1 with the overexpressed endogenous *hfq* under control of the *tac* promoter; US6-1/P*tac-srmB*^33A^: *N. pentaromativorans* US6-1 with the heterologously expressed *srmB* from *Glaciecola* sp. 33A under control of the *tac* promoter; US6-1/P*tac-hfq*^33A^: *N. pentaromativorans* US6-1 with the heterologously expressed *hfq* from *Glaciecola* sp. 33A under control of the *tac* promoter; and US6-1/P*tac-deaD*^33A^: *N. pentaromativorans* US6-1 with the heterologously expressed *deaD* from *Glaciecola* sp. 33A under control of the *tac* promoter. PHE: phenanthrene. Unless otherwise specified, the expression of genes under control of the *tac* promoter was induced by 0.1 mM IPTG. Significant difference: ns, *P* > 0.05; *, *P* < 0.05; **, *P* < 0.01.

The cold shock response is a comprehensive molecular adaptive reaction that occurs when cells experience a sudden drop in temperature. Bacteria regulate global gene expression to counteract the impacts of low temperatures by changing the DNA structure, transcription processes, RNA metabolism, and translation recovery (27). In this study, 18°C was selected as the cold temperature for testing. The results showed that the overexpression of endogenous cold shock response proteins (Table S4) enhanced the cold tolerance of *N. pentaromativorans* US6-1. In particular, the overexpression of *deaD* and *hfq* exhibited the most significant improvement in cold resistance (Fig. 4A and B). The OD_600_ of US6-1/P*tac-deaD* and US6-1/P*tac-hfq* at 60 h increased to 1.15 times and 1.23 times that of the control strain (US6-1/P*tac*), respectively, and the residual phenanthrene of US6-1/P*tac-deaD* and US6-1/P*tac-hfq* at 12 h was 63.8% and 75.0% that of the control strain, respectively. It has been reported that these genes participate in ribosome biogenesis and assembly processes under low-temperature conditions (28–30). Therefore, it was hypothesized that impaired ribosome assembly may represent the primary contributor to the defects of *N. pentaromativorans* US6-1 at low temperatures, and the overexpression of genes involved in ribosome assembly may relieve these cold-associated defects. To further examine the genetic elements of ribosome assembly, the evolutionary relationship between *N. pentaromativorans* US6-1 and psychrophilic bacteria was analyzed by drawing a phylogenetic tree (Fig. S5). Most of the ribosome assembly factors exhibited high similarity between *N. pentaromativorans* US6-1 and *Glaciecola* sp. 33A. In addition, unlike *Glaciecola* sp. 33A, *N. pentaromativorans* US6-1 naturally lacks SrmB, an important cold-associated assembly factor. It has been reported that the overexpression of *deaD* can partially relieve the defects of *srmB*-null-caused ribosome biogenesis at low temperatures (31). Therefore, *deaD*, *srmB*, and *hfq* from *Glaciecola* sp. 33A were selected as candidate cold-resistant factors (Table S4) and heterologously expressed in *N. pentaromativorans* US6-1. Notably, among these three candidate factors, SrmB gave *N. pentaromativorans* US6-1 the best cold resistance at 18°C. The OD_600_ at 60 h and the residual phenanthrene at 12 h of US6-1/P*tac-srmB*^33A^ were 1.30 times and only 43.7% that of the control strain (US6-1/P*tac*), respectively (Fig. 4C and D). Therefore, the SrmB from *Glaciecola* sp. 33A was selected as an ideal cold-resistant genetic element for the construction of a customized cold-resistant system in *N. pentaromativorans* US6-1. SrmB is a ribosome assembly factor involved in ribosome biogenesis at low temperatures (32). It is assumed that SrmB accelerates the ribosome biogenesis efficiency at low temperatures and thus reduces the damage to cells caused by low temperatures.

### Control of the cold-resistant system for cold-responsive regulation in *N. pentaromativorans* US6-1

**Fig 5:**
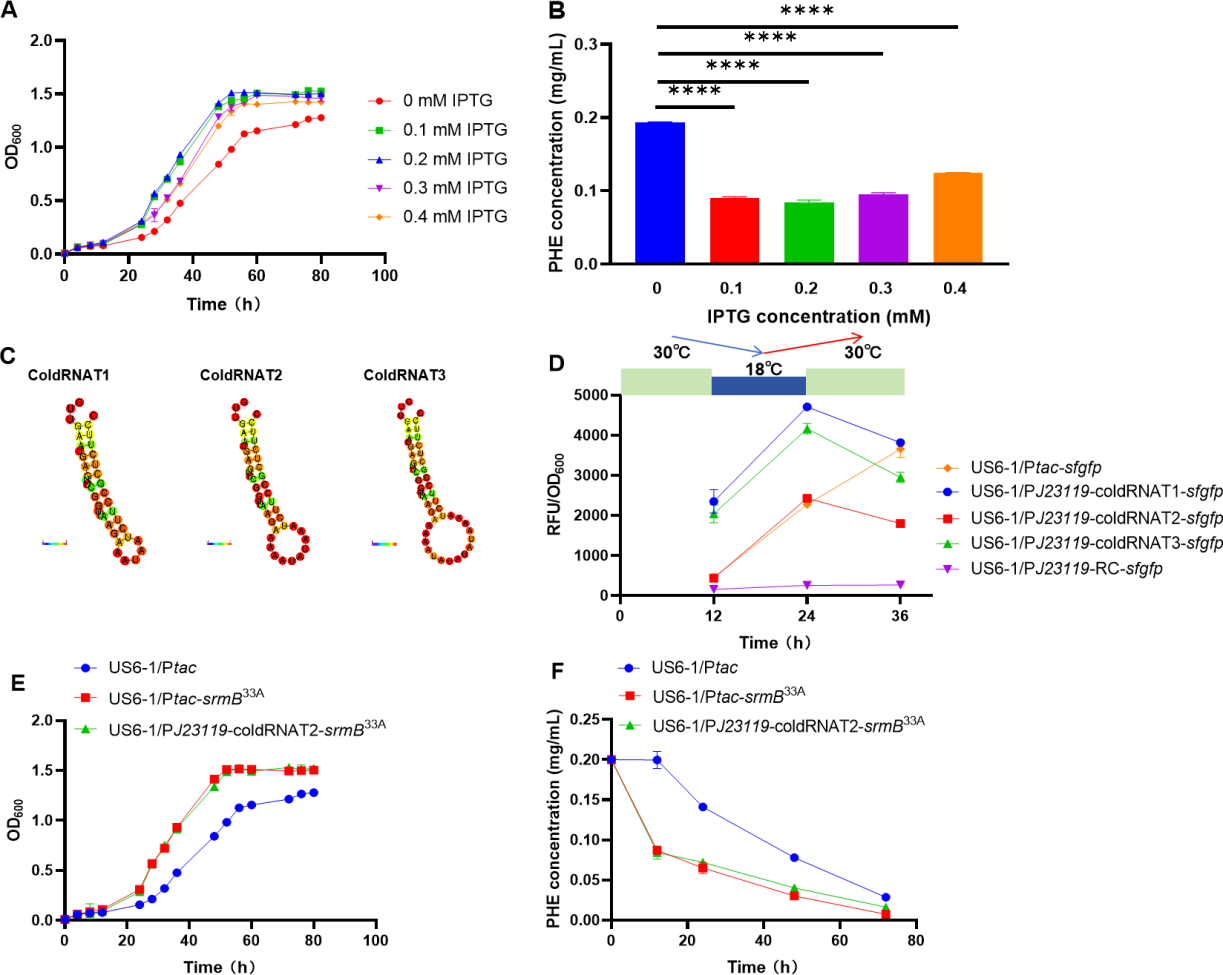
Construction and evaluation of cold-induced genetic switches. (**A and B**) The inducible promoter P*tac* was used to control the expression of cold-resistant element *srmB* from *Glaciecola* sp. 33A (*srmB*^33A^) in *N. pentaromativorans* US6-1 (US6-1/P*tac-srmB*^33A^) with different IPTG concentrations and effects of the *srmB*^33A^ expression on the cell growth (**A**) and phenanthrene degradation (**B**) at 18°C for 12 h were evaluated. Cells were cultured in P5Y3 medium for measurement of the growth curve, whereas they were cultured in a P5Y3 medium with 0.2 mg/mL phenanthrene for measurement of the phenanthrene degradation curve. (**C**) Predicted structure of the candidate cold-induced RNATs (cold-RNATs). (**D**) Evaluation of cold-induced switches constructed by combining cold-RNATs with constitutive promoter P*J23119*. The reporter gene *sfgfp* was used to explore the optimal cold-induced switch for control of the cold-tolerant system, and the *tac* promoter was used as the control of the chemical IPTG-induced switch for comparison. Cells were first cultured at 30°C for 12 h, then transferred to 18°C (0.2 mM IPTG was immediately added to the control of chemical IPTG-induced switch to induce the expression of *sfgfp*) for 12 h, and finally transferred back to 30°C for another 12 h. US6-1/P*tac-sfgfp: N. pentaromativorans* US6-1 with the heterologously expressed *sfgfp* under control of the *tac* promoter; US6-1/P*J23119*-coldRNAT1-*sfgfp* to US6-1/P*J23119*-coldRNAT3-*sfgfp: N. pentaromativorans* US6-1 with the heterologously expressed *sfgfp* under control of constitutive promoter P*J23119* combined with different cold-induced RNATs (RNAT1 to RNAT3); US6-1/P*J23119*-RC-*sfgfp: N. pentaromativorans* US6-1 with the heterologously expressed *sfgfp* under control of constitutive promoter P*J23119* combined with a RNase E-recognized cleavage site (RC), which allows RNase E to degrade mRNA and inhibiting the expression of *sfgfp* at 18°C. (**E and F**) Effects of optimized cold-induced switch and chemical IPTG-inducted switch on the cell growth (**E**) and phenanthrene degradation (**F**) at 18°C. Cells were cultured in P5Y3 medium for measurement of the growth curve, whereas they were cultured in a P5Y3 medium with 0.2 mg/mL phenanthrene for measurement of the phenanthrene degradation curve. US6-1/P*tac*: wild-type strain of *N. pentaromativorans* US6-1 containing blank plasmid vector P*tac*; US6-1/P*tac-srmB*^33A^: *N. pentaromativorans* US6-1 with the heterologously expressed *srmB*^33A^ under control of the *tac* promoter; US6-1/P*J23119*-coldRNAT2-*srmB*^33A^: *N. pentaromativorans* US6-1 with the heterologously expressed *srmB*^33A^ under control of constitutive promoter P*J23119* combined with cold-induced switch coldRNAT2. PHE: phenanthrene. Unless otherwise specified, the expression of genes under control of the *tac* promoter was induced by 0.2 mM IPTG. Significant difference: ****, *P* < 0.0001.

To investigate the optimal expression level of cold-responsive genes at 18°C, the inducible promoter P*tac* was employed to manipulate the gene expression using varying IPTG concentrations. The results showed that under 0.2 mM IPTG, the strain US6-1/P*tac-srmB*^33A^ at 18°C grew the best (Fig. 5A). The residual phenanthrene of US6-1/P*tac-srmB*^33A^ after 12 h of degradation at 18°C was also the most minimal under 0.2 mM IPTG, which is only 41.1% of that of the control strain (US6-1/P*tac*) (Fig. 5B). These findings indicated that the P*tac* promoter achieved the best expression intensity of *srmB*^33A^ for *N. pentaromativorans* US6-1 at 18°C under 0.2 mM IPTG, providing an ideal “ON” state for a cold-induced switch.

To establish a cold-responsive regulation system in *N. pentaromativorans* US6-1, a low temperature-triggered switch was engineered to regulate the expression of cold-responsive genes. Unlike the heat-RNATs in the designed heat-induced switch, the cold RNA-thermosensors (cold-RNATs) used in this study contained a RNase E-recognized cleavage site (RC) in the 5’ untranslated region of the target gene, which was the same as the RC reported in *E. coli* (33) according to the RNase E motif analysis (Fig. S6). At low temperatures, the RC is enclosed within a stem-loop structure, allowing for smooth gene expression. On contrary, the stem-loop structure unfolds at high temperatures, leading to mRNA degradation and the cessation of gene expression. In the cold-resistant system, P*J23119* was selected as the constitutive promoter, and three cold-RNATs (coldRNAT1, coldRNAT2, and coldRNAT3) (Table S5) were designed using the two-state melting hybridization function of DINAMelt software (Fig. 5C), and sfGFP was utilized as the reporter to assess the functionality of various switches. To verify the function of the RC sequence in preventing gene expression, this study first combined the constitutive promoter J*23119* with the RC sequence to regulate the expression of *sfgfp* (US6-1/P*J23119*-RC-*sfgfp*). The results indicated that the expression of *sfgfp* (the relative fluorescence intensity) was significantly inhibited after the insertion of the RC sequence, confirming that the addition of RC sequence prevented gene expression (Fig. 5D). The results further showed that the relative fluorescence intensity of US6-1/P*J2311*9-coldRNAT2-*sfgfp* exhibited the closest approximation to that of US6-1/P*tac-sfgfp* under 0.2 mM IPTG at 18°C (Fig. 5D). Therefore, P*J2311*9-coldRNAT2 was selected as the environmental cold-responsive switch to regulate the expression of *srmB*^33A^ in *N. pentaromativorans* US6-1 at low temperatures. Accordingly, the cold-tolerant and -responsive strain US6-1/P*J2311*9-coldRNAT2-*srmB*^33A^ was constructed. Notably, the OD_600_ and residual phenanthrene of US6-1/P*J2311*9-coldRNAT2-*srmB*^33A^ at 18°C after 12 h of incubation was 1.29 times and 41.4% that of the control strain (US6-1/P*tac*), respectively (Fig. 5E and F). In addition, the optimized cold-induced and -responsive switch P*J2311*9-coldRNAT2 exhibited similar performance compared with the chemical IPTG-induced switch.

### Construction and performance of the TAS circuit

**Fig 6:**
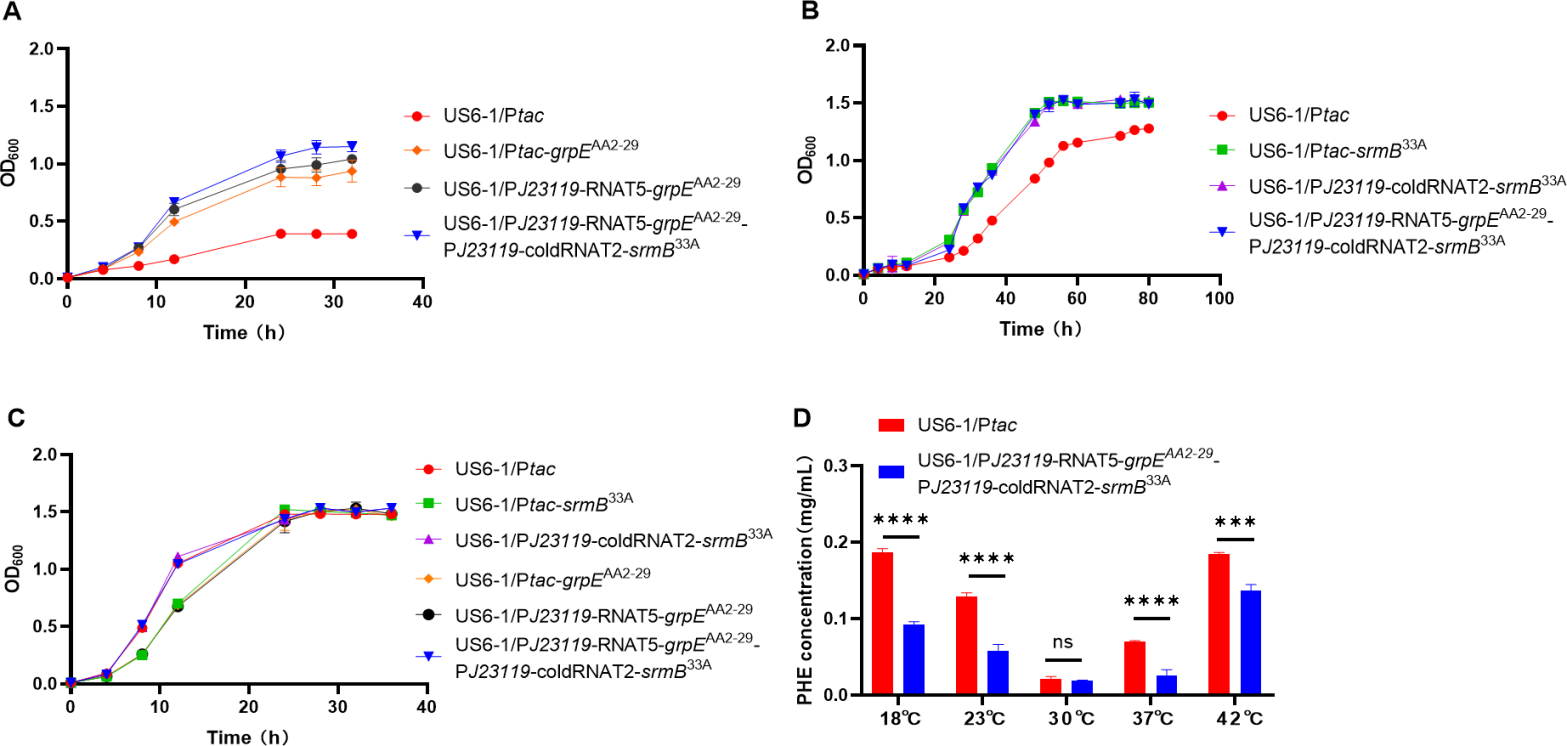
Construction and evaluation of the temperature adaptation system (TAS) circuit. Growth curves of the strains at 42°C (**A**), 18°C (**B**), and 30°C (**C**). Cells were cultured in P5Y3 medium. (**D**) Phenanthrene (PHE) degradation of the strains at 18°C, 23°C, 30°C, 37°C, and 42°C for 12 h. Cells were they were cultured in a P5Y3 medium with 0.2 mg/mL phenanthrene. US6-1/P*tac*: wild-type strain of *N. pentaromativorans* US6-1 containing blank plasmid vector P*tac*; US6-1/P*tac-grpE*^AA2-29^: *N. pentaromativorans* US6-1 with the heterologously expressed *grpE* from *T. thermophilus* AA2-29 (*grpE*^AA2-29^) under control of the *tac* promoter; US6-1/P*tac-srmB*^33A^: *N. pentaromativorans* US6-1 with the heterologously expressed *srmB* from *Glaciecola* sp. 33A (*srmB*^33A^) under control of the *tac* promoter; US6-1/P*J23119*-RNAT5-*grpE*^AA2-29^: *N. pentaromativorans* US6-1 with the heterologously expressed *grpE*^AA2-29^ under control of constitutive promoter P*J23119* combined with heat-induced switch RNAT5 (P*J23119*-RNAT5); US6-1/P*J23119-*coldRNAT2-*srmB*^33A^: *N. pentaromativorans* US6-1 with the heterologously expressed *srmB*^33A^ under control of constitutive promoter P*J23119* combined with cold-induced switch coldRNAT2 (P*J23119*-coldRNAT2); and US6-1/P*J23119*-RNAT5-*grpE*^AA2-29^-P*J23119*-coldRNAT2-*srmB*^33A^: *N. pentaromativorans* US6-1 with expressing a temperature adaptation system (TAS) constructed by combining P*J23119*-RNAT5-*grpE*^AA2-29^ with P*J23119*-coldRNAT2-*srmB*^33A^. Unless otherwise specified, the expression of genes under control of the *tac* promoter was induced by 0.2 mM IPTG. Significant difference: ns, *P* > 0.05; ***, *P* < 0.001; ****, *P* < 0.0001.

To broaden the working temperature range of chassis cells from high temperatures to low temperatures, the TAS circuit P*J2311*9-RNAT5-*grpE*^AA2-29^-P*J2311*9-coldRNAT2-*srmB*^33A^ (Fig. S7) was created by merging the customized thermotolerant system with the customized cold-resistant system. With the assistance of the TAS circuit, the OD_600_ of US6-1/P*J2311*9-RNAT5-*grpE*^AA2-29^-P*J2311*9-coldRNAT2-*srmB*^33A^ was 2.68 times that of the control strain (US6-1/P*tac*) at 42°C for 24 h (Fig. 6A), 1.29 times that of the control strain (US6-1/P*tac*) at 18°C for 60 h (Fig. 6B), and similar to that of the control strain (US6-1/P*tac*) at 30°C (Fig. 6C). In addition, after 12 h, the residual phenanthrene of US6-1/P*J2311*9-RNAT5-*grpE*^AA2-29^-P*J2311*9-coldRNAT2-*srmB*^33A^ was 49.4% that of the control strain (US6-1/P*tac*) at 18°C, 45.3% that of the control strain (US6-1/P*tac*) at 23°C, 37.1% that of the control strain (US6-1/P*tac*) at 37°C, 73.8% that of the control strain (US6-1/P*tac*) at 42°C, and similar to that of the control strain (US6-1/P*tac*) at 30°C (Fig. 6D). Altogether, these results demonstrated that the constructed genetic circuit TAS could effectively respond to temperature changes and enhance the stability of chassis cells at adverse temperatures, thus broadening the working temperature for *N. pentaromativorans* US6-1. Additionally, to investigate whether similar effects are observed in other phenanthrene-degrading bacteria when heat- and cold-tolerance mechanisms are introduced, the TAS circuit was introduced into *Croceicoccus naphthovorans* PQ-2, another phenanthrene-degrading bacterium (34). The results in Fig. S8 showed that this TAS circuit can also promote the cell growth and phenanthrene degradation of *C. naphthovorans* PQ-2 when exposed to 42°C and 18°C, providing broader insights into the application of the TAS circuit across different bacterial strains.

## DISCUSSION

PAHs are persistent pollutants known for their extensive biological toxicities (35). The efficient *in situ* bioremediation of PAH-contaminated sites has attracted increasing attention. However, the low survivability and tolerance of PAH-degrading microbes under harsh conditions, especially under varying temperatures, represent a bottleneck for the effective application of microbial remediation. The rapid development of synthetic biology has made it possible to enhance the survivability and adaptability of microbes under harsh conditions (36). This study, for the first time, presents a temperature-responsive system, TAS, created by integrating a customized thermotolerant system with a customized cold-resistant system (Fig. 1). The introduction of the TAS circuit into the PAH-degrading bacterium *N. pentaromativorans* US6-1 ensured the robust biological activity of cells from 18°C to 42°C, offering great potential for the efficient *in situ* bioremediation of PAHs under harsh conditions and varying temperatures.

In practical applications, the biological activity of microbial cells is significantly influenced by the environmental temperature. Precise modification of stress resistance in microbial chassis cells aims to enhance their growth and metabolism efficiency during temperature fluctuations. Bacteria typically possess inherent mechanisms for heat and cold tolerance and may have had weaker innate tolerance to these conditions. The results of this study revealed that the overexpression of endogenous heat shock proteins and cold shock response proteins in *N. pentaromativorans* US6-1 promoted the growth and PAH degradation of cells when exposed to high and low temperatures, respectively. In theory, extremophiles offer a rich reservoir of stress-resistance elements that likely exhibit better functions than those in mesophilic microbes (37). It has been found that stress-resistance elements that exist both in extremophiles and mesophilic microbes and show high homologies were well expressed in mesophilic microbes (12, 38). Therefore, the evolutionary relationship between extremophiles and mild microbes should be analyzed to improve the biocompatibility of genetic elements from extremophiles for applications in mild microbes. The phylogenetic analyses conducted in the present study indicated that the thermotolerant element *grpE* from *T. thermophilus* AA2-29 and the cold-resistant element *srmB* from *Glaciecola* sp. 33A may be crucial for temperature stress resistance and suitable for engineering *N. pentaromativorans* US6-1. The subsequent introduction of exogenous *grpE*^AA2-29^ and *srmB*^33A^ into *N. pentaromativorans* US6-1 exhibited biocompatibility and further improved the adaption of cells to temperature stress. Therefore, bioinformatics-aided analyses could be important for the selection and application of stress-resistant components from extremophiles in mild microbial chassis.

The functional and appropriate expression of temperature-resistant genetic elements is crucial for cells to adapt to fluctuating temperatures and conserve cell resources (15). Therefore, the precise regulation of temperature-resistant element expression is essential for temperature stress tolerance and the minimization of side effects. IPTG is a portable and useful chemical inducer that can precisely and tightly regulate gene expression (39). However, the IPTG induction strategy is limited by obvious drawbacks, including the need for human supervision, host toxicity, and the metabolic burden imposed by the presence of IPTG (40). Therefore, temperature-responsive regulation switches are more promising than IPTG-induced chemical switches in practical applications (41). In this study, base pairing-associated heat-RNATs and RNase E-based cold-RNATs were employed to autonomously regulate the expression of heat-responsive elements and cold-responsive elements, respectively. Furthermore, the introduction of combined heat-RNATs and cold-RNATs (the TAS circuit) into *N. pentaromativorans* US6-1 enabled the engineered cells to express the desired temperature-responsive elements upon sensing temperature fluctuations. Despite the advancements in the TAS circuit, there are still some limitations. The main limitations include the following: (i) the operating temperature range of *N. pentaromativorans* US6-1 still needs to be further expanded; (ii) the phenanthrene degradation efficiency of *N. pentaromativorans* US6-1 at different temperatures still needs to be further improved; and (iii) the modification should be customized based on actual application scenarios.

In the future, the TAS circuit and similar systems can be designed for other chassis, which could provide broader insights into the application of the system across different bacterial strains. With the richness of the HSP and CIP libraries, the temperature tolerance of cells is expected to be significantly extended to higher levels. Furthermore, customized TAS circuits could provide benefits to different metabolic pathways based on their specific properties and the requirements of the production processes in non-isothermal temperature fermentation. Moreover, the designed idea can be applied to construct a super chassis that exhibits tolerance to multiple stresses derived from pH levels, oxygen concentrations, and osmotic pressure (42).

In summary, this work demonstrated the potential application of synthetic biology to enhance the temperature-resistant abilities of chassis cells. In addition to diminishing the need for precise temperature control in bioremediation, this approach lowered the cost associated with biotransformation, representing a significant milestone in the advancement of applied microbiology and industrial biotechnology.

## MATERIALS AND METHODS

### Bacterial strains, plasmids, growth conditions, and primers

All bacterial strains and plasmids utilized in this study are listed in Table S6. *E. coli* WM3064 was cultured in Luria–Bertani (LB) medium at 37°C, whereas *N. pentaromativorans* US6-1 was cultured in P5Y3 medium at 30°C. For the biodegradation assay, *N. pentaromativorans* US6-1 was cultivated at 30°C in a marine minimal medium supplemented with phenanthrene (200  mg/L) as the sole carbon source (43). If necessary, the media were supplied with chemicals at the following concentrations: 5.7  µg/mL of 2,6-diaminopimelic acid (DAP), 50  µg/mL of kanamycin (Km), and 0.1 mM of IPTG. All primers used in common molecular experiments are listed in Table S7.

### Quantitative real-time polymerase chain reaction (RT-qPCR)

RT-qPCR analysis was performed to determine the transcription levels of the genes. The sequences of the genes are shown in Table S1. The *N. pentaromativorans* US6-1 strain was cultured overnight to the stationary phase. The extraction of total RNA was conducted using the RNAiso Plus kit (TaKaRa, Dalian, China) according to the manufacturer’s guidelines. The cDNA was synthesized using HiScript IIQ Select RT SuperMix (Vazyme, Nanjing, China). RT-qPCR analysis was performed utilizing the CFX Connect real-time PCR detection system (Bio-Rad, USA). The cycle threshold (*C*_T_) values for each gene were normalized against the *C*_T_ values of the 16S rRNA gene (44).

### Construction of the overexpression strains

The broad-host-range plasmid pHGE-P*tac* (45) was employed to overexpress the thermotolerant and cold-resistant elements. The target gene fragment was amplified using primers (Table S7) and then connected with the plasmid vector through the one-step cloning method according to the manufacturer’s instructions (Vazyme, Nanjing, China). The constructed plasmid was subsequently transformed into *E. coli* WM3064 using the basic molecular method, and its authenticity was confirmed based on DNA sequencing (Vazyme, Nanjing, China). The plasmid was then transferred to the corresponding wild-type strain via conjugation for phenotypic analysis using IPTG.

### Design of temperature-induced switches and prediction of the secondary structure

Paired hairpin sequences were deemed as discrete segments, and the two-state melting hybridization function provided from the website (http://www.unafold.org/) was employed to design RNAT autonomously, which was based on the specified melting temperature. The complete RNA sequence was subsequently inputted into RNAFold (http://rna.tbi.univie.ac.at/cgi-bin/RNAWebSuite/RNAfold.cgi) for structural prediction, and the corresponding images were then generated as a reference.

### Determination of the fluorescence values

As for the establishment of the *sfgfp* reporting system, the initial step involved the integration of the *sfgfp* gene into the plasmid pHGE-P*tac*. The tac promoter (P*tac*) was subsequently substituted with the synthetic promoter (BBa_*J23119*) sourced from the Anderson promoter library. This substitution was conducted using the one-step cloning method according to the manufacturer’s instructions (Vazyme, Nanjing, China). Furthermore, the temperature-induced switch was inserted at the 5’UTR position through overlapping PCR. The plasmid was then transferred to the corresponding wild-type strain via conjugation for phenotypic analysis.

The *sfgfp* reporting system strains were cultured overnight in P5Y3 broth until an OD_600_ value of 1.0. Subsequently, the overnight cultures were diluted 1:100 in 50 mL of fresh P5Y3 broth. Following 12 h of incubation at 30°C and 180 rpm, the temperature was either adjusted to 42°C or maintained at 18°C for an additional 12 h period, prior to returning the temperature to 30°C for continued cultivation. Subsequently, fluorescence measurement was conducted. In brief, aliquots of 200 µL cultures were dispensed into 96-well black microplates, and absorbance and fluorescence were measured. The absorbance measurement was employed at 600 nm. Normalized fluorescence units to OD_600_ (normalized RFU) were accordingly calculated. For detection, the excitation wavelength and the emission wavelength were set to 488 nm and 520 nm, respectively.

### Measurement of the residual phenanthrene

The residual phenanthrene presented in the medium was determined by the utilization of high-performance liquid chromatography (HPLC) techniques (34). Initially, the samples were extracted using ethyl acetate and then evaporated to concentrate the analytes. Subsequently, the extracts were dissolved into methanol for effective solubilization, followed by filtration to remove any particulate matter. These prepared samples were then subjected to HPLC analysis.

ShimNex S-C18-PAH chromatography column was used for HPLC analysis. The HPLC conditions were set as follows: the mobile phase was a mixture of acetonitrile and water in a volume ratio of 60:40; the flow rate was maintained at 1.5 mL/min; the injection volume was 5 µL; the column temperature was 25°C; and the detection wavelength was set to 254 nm.

### Statistical analysis

Unless otherwise specified, all the results presented were achieved from three independently repeated experiments. Student’s *t*-test was conducted for pairwise comparison. Values of the data were displayed as means and standard errors of the mean.

## SUPPLEMENTAL MATERIAL

Supplemental material aem.01484-24-s0001.pdf Figures S1 to S8; Tables S1 to S7.

ASM does not own the copyrights to Supplemental Material that may be linked to, or accessed through, an article. The authors have granted ASM a non-exclusive, world-wide license to publish the Supplemental Material files. Please contact the corresponding author directly for reuse.
