# Supplementary material for: Temperature-responsive regulation of the polycyclic aromatic hydrocarbon-degrading mesophilic bacterium Novosphingobium pentaromativorans US6-1 with a temperature adaptation system
Source: Appl Environ Microbiol. 2024 Dec 12;91(1):e01484-24. doi: 10.1128/aem.01484-24 (PMC11784078; doi:10.1128/aem.01484-24)
Supplement: Supplemental material — Figures S1 to S8; Tables S1 to S7. [file aem.01484-24-s0001.pdf]

Supplementary Materials

Temperature-responsive regulation of polycyclic aromatic hydrocarbon-degrading mesophilic bacterium *Novosphingobium pentaromativorans* US6-1 with temperature adaptation system

Zhuangzhuang Liu[a], Xinran Liu[a], Haiyan Huang[a], Feifei Cao[b], Qiu Meng[a], Tingheng Zhu[a], Jianhua Yin[a], Xiaofei Song[a]*, Zhiliang Yu[a]*

[a]College of Biotechnology and Bioengineering, Zhejiang University of Technology, Hangzhou 310014, Zhejiang Province, China

[b]Hangzhou Chuhuan Science and Technology Co., Ltd., Hangzhou 310015, Zhejiang Province, China

*Corresponding authors:

zlyu@zjut.edu.cn (Zhiliang Yu); xiaofei@zjut.edu.cn (Xiaofei Song)

Running title: Engineered temperature adaption system

Keywords: Temperature-responsive system, Synthetic biology, Polycyclic aromatic hydrocarbons, Biodegradation, *Novosphingobium pentaromativorans*

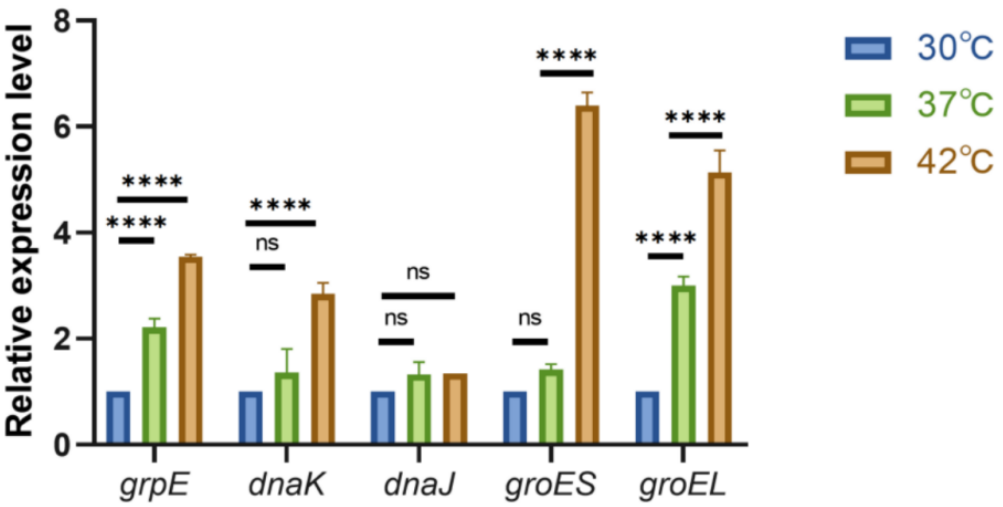

FIG S1. Transcription levels of heat shock protein-encoding genes at different temperatures. The wild type strain of *Novosphingobium pentaromativorans* US6-1 was cultured in P5Y3 medium at 30 ℃, 37 ℃, and 42 ℃, and the changes in transcription levels of *grpE*, *dnaK*, *dnaJ*, *groEL*, and *groES* at different temperatures were measured. Information of heat shock protein-encoding genes was displayed in supplementary Table S1. Significant difference: ns, P> 0.05; *, P< 0.05; ****, P< 0.0001.

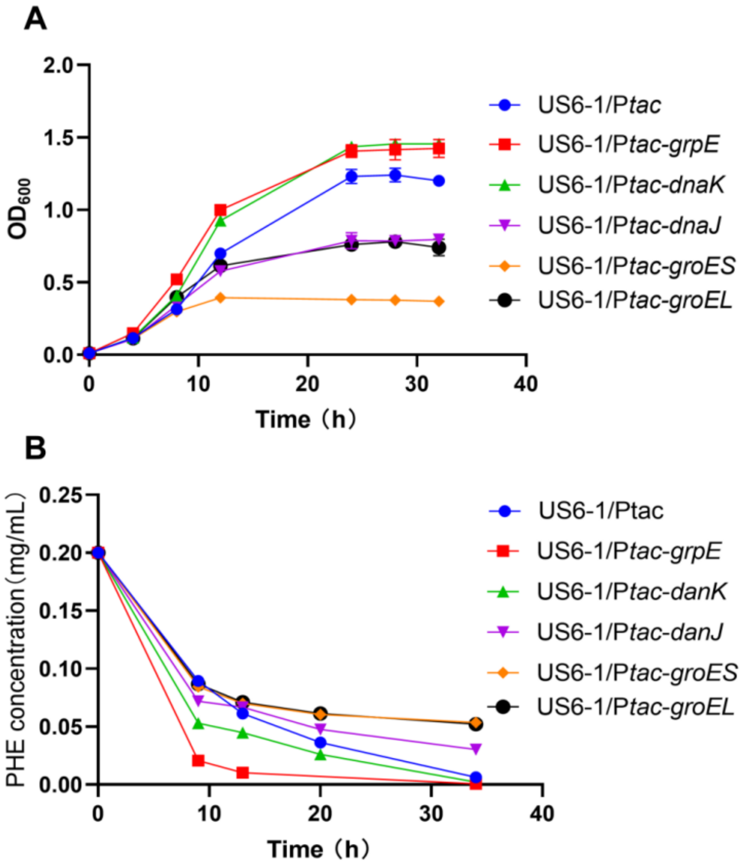

FIG S2. Cell growth (A) and phenanthrene (PHE) degradation (B) of heat shock protein-overexpressed strains at 37 ℃. For growth curve, cells were cultured in P5Y3 medium. For residual phenanthrene curve, cells were cultured in P5Y3 medium with 0.2 mg/mL phenanthrene. US6-1/P*tac*: wild type strain of *N. pentaromativorans* US6-1 containing blank plasmid vector P*tac*; US6-1/P*tac-grpE*: *N. pentaromativorans* US6-1 with the overexpressed endogenous *grpE* under control of the *tac* promoter; US6-1/P*tac-dnaK*: *N. pentaromativorans* US6-1 with the overexpressed endogenous *dnaK* under control of the *tac* promoter; US6-1/P*tac-danJ*: *N. pentaromativorans* US6-1 with the overexpressed endogenous *danJ* under control of the *tac* promoter; US6-1/P*tac-groEL*: *N. pentaromativorans* US6-1 with the overexpressed endogenous *groEL* under control of the *tac* promoter; US6-1/P*tac-groES*: *N. pentaromativorans* US6-1 with the overexpressed endogenous *groES* under control of the *tac* promoter. Information of heat shock protein-encoding genes was displayed in supplementary Table S1. The expression of genes was induced by 0.1 mM IPTG.

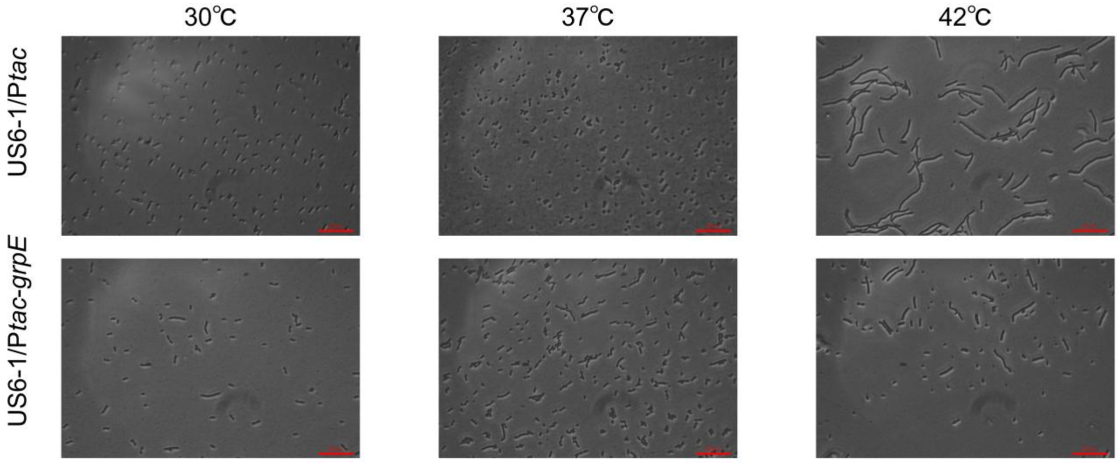

FIG S3. Morphology of the strains at different temperatures. Cells were cultured in solid P5Y3 medium at 30 ℃, 37 ℃, and 42 ℃. US6-1/P*tac*: wild type strain of *N. pentaromativorans* US6-1 containing blank plasmid vector P*tac*; US6-1/P*tac-grpE*: *N. pentaromativorans* US6-1 with the overexpressed endogenous *grpE* under control of the *tac* promoter. Information of *grpE* was displayed in supplementary Table S1. The expression of genes was induced by 0.1 mM IPTG. Scale bar: 10 μm.

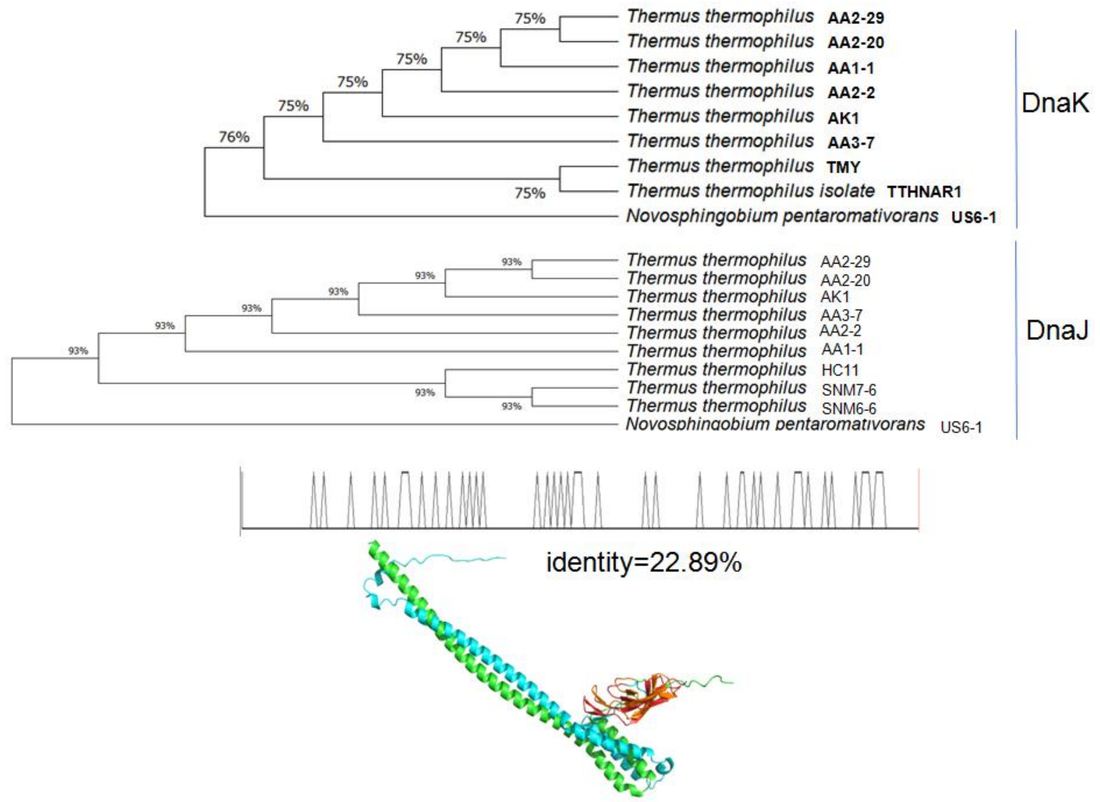

FIG S4. Comparison of heat shock proteins between mesophilic bacterium *Novosphingobium pentaromativoran*s US6-1 and thermophilic strains. Phylogenetic tree of heat shock proteins DnaK and DnaJ was constructed from strain US6-1 and thermophilic bacteria. An unrooted Neighbor-Joining phylogenetic tree of proteins in the SSN was constructed using the MUSCLE alignment method and MEGA11. The evolutionary history was represented by the bootstrap consensus tree inferred from 1000 replicates. Numbers at nodes represent the bootstrap values. Comparison of the three-dimensional structure of GrpE between *N. pentaromativorans* US6-1 and *Thermus thermophilus* AA2-29 (*N. pentaromativorans* US6-1 in blue and *T. thermophilus* AA2-29 in green) was displayed at bottom with identity. Information of *grpE* was displayed in supplementary Table S1.

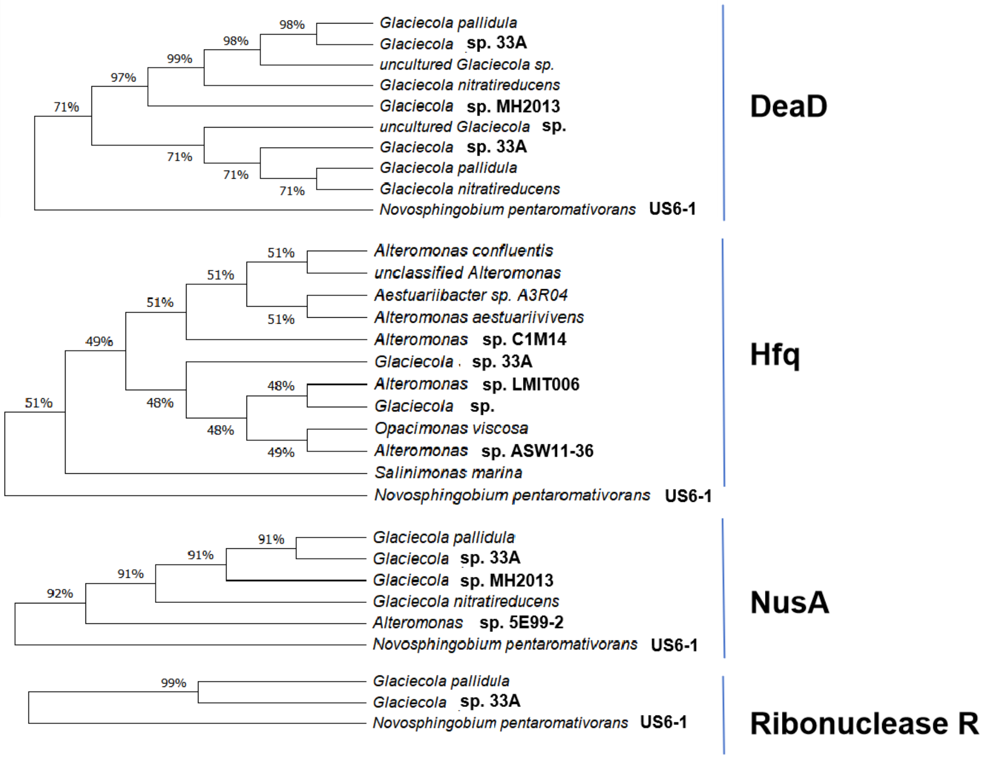

FIG S5. Phylogenetic tree of ribosomal generating factor related genes. An unrooted Neighbor-Joining phylogenetic tree of proteins in the SSN was constructed using the MUSCLE alignment method and MEGA11. The evolutionary history was represented by the bootstrap consensus tree inferred from 1000 replicates. Numbers at nodes represent the bootstrap values. Information of cold shock protein-encoding genes was displayed in supplementary Table S4.

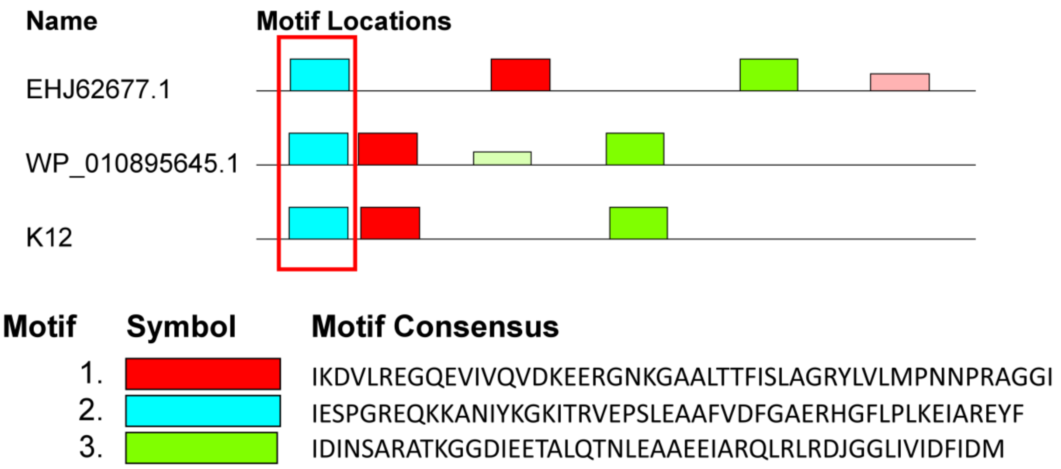

FIG S6. Analyses of RNase E motif. Motif of RNase E protein from *Novosphingobium pentaromativorans* US6-1 strain, *Escherichia coli* K12, and *Pseudomonas aeruginosa* PAO1 was compared. The sites of RNA sequences recognized by RNase E were marked in blue.

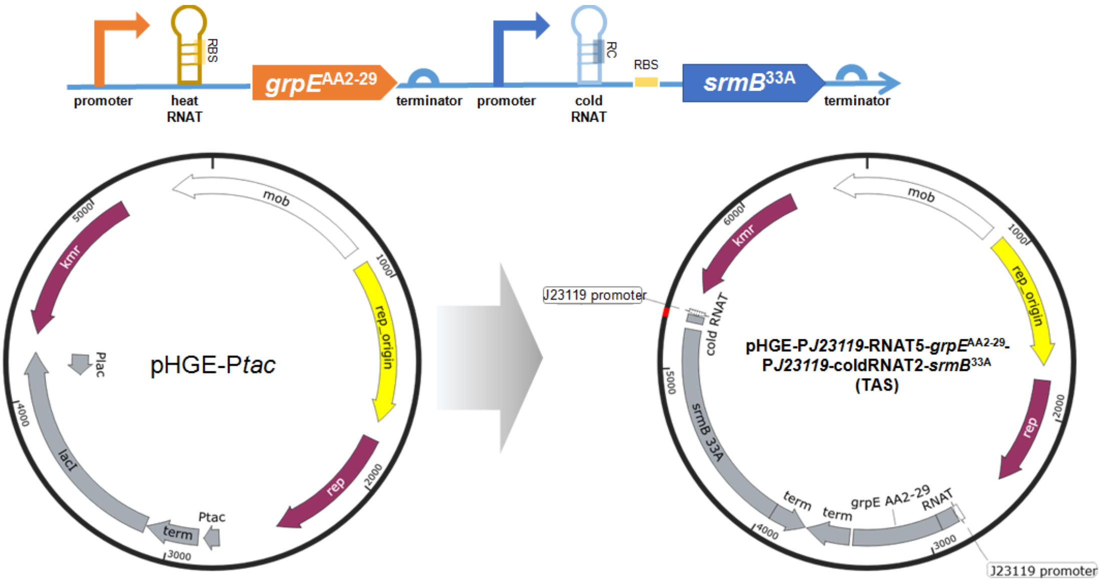

FIG S7. Plasmid map. The original plasmid of pHGE-P*tac* was displayed in left; the recombinant plasmid of pHGE-P*J23119*-RNAT5-*grpE*$^{AA2\text{-}29}$-P*J23119*-coldRNAT2-*srmB*^{33A} constructed by introducing the temperature adaptation system (TAS) into pHGE-P*tac* was displayed in right.

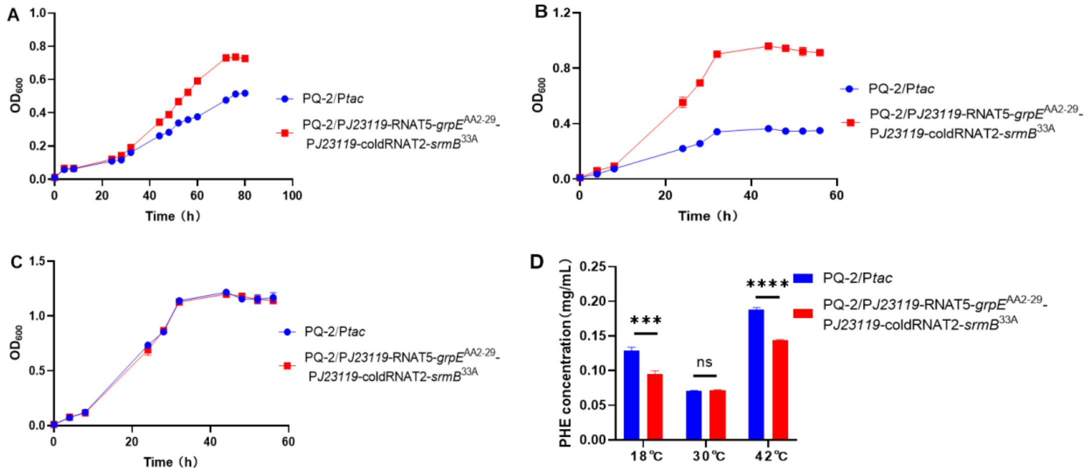

FIG S8. Evaluation of the temperature adaptation system (TAS) circuit in *Croceicoccus naphthovorans* PQ-2. Growth curves of the strains at **42 ℃ (A)**, **18 ℃ (B)**, and **30 ℃ (C)**. Cells were cultured in P5Y3 medium. (D) Phenanthrene (PHE) degradation of the strains at 18 ℃, 30 ℃, and 42 ℃ for 60 h. Cells were cultured in P5Y3 medium with 0.2 mg/mL phenanthrene. PQ-2/P*tac*: wild type strain of *C. naphthovorans* PQ-2 containing blank plasmid vector P*tac*; PQ-2/P*J23119*-RNAT5-*grpE*$^{AA2-29}$-P*J23119*-coldRNAT2-*srmB*^{33A}: *C. naphthovorans* PQ-2 with expressing a temperature adaptation system (TAS) constructed by combining P*J23119*-RNAT5-*grpE*$^{AA2-29}$ with P*J23119*-coldRNAT2-*srmB*^{33A}. *C. naphthovorans* PQ-2 is a phenanthrene-degrading bacterium. Unless otherwise specified, the expression of genes under control of the *tac* promoter was induced by 0.2 mM IPTG. Significant difference: ns, P> 0.05; ***, P< 0.001; ****, P< 0.0001.

Supplementary Table S1. Information of heat-resistant genetic elements.

Gene	Protein	Source	Locus tag
grpE	Nucleic acid exchange factor GrpE	*N. pentaromativorans* US6-1	NSU_4359
dnaK	Chaperone protein DnaK (HSP70)	*N. pentaromativorans* US6-1	NSU_4355
dnaJ	Chaperone protein DnaJ	*N. pentaromativorans* US6-1	NSU_4354
groEL	Chaperonin GroEL (HSP60)	*N. pentaromativorans* US6-1	NSU_2343
groES	Co-chaperonin GroES	*N. pentaromativorans* US6-1	NSU_2344
grpE$^{AA2\text{-}29}$	Nucleic acid exchange factor GrpE	*Thermus thermophilus* AA2-29	TthAA229_15450

Supplementary Table S2. Sequences of designed heat-RNATs.

Name	Anti-RBS	Loop region	RBS	Tm (℃)	ΔG (kcal/mol)
RNAT1	CACGGTGAA CCTCTCTCCG CTA	AAAATATA TATAAAA	AGGAGAGA ATTCAATG	41.8	-11.3
RNAT2	CATTCAATTC TCTCGTA	AAAATATA TATAAAA	AGGAGAGA ATTCAATG	42.0	-10.9
RNAT3	CATTACAATT CTCTCGTA	AAAATATA TATAAAA	AGGAGAGA ATTCAATG	40.3	-11.0
RNAT4	CATTGAATTC GTGGGTCCTA	AAAATATA TATAAAA	AGGAGAGA ATTCAATG	40.5	-11.1

Note: RBS (ribosome binding site) was marked by underline, and the start codon was shown in green. This table shows RNA sequences designed from scratch, and Tm value and G value were calculated by the UNAFold Web Server (http://www.unafold.org/).

Supplementary Table S3. Sequences of heat-RNATs and constitutive promoter P*J23119* from the iGEM Registry.

Name	Type and source	DNA sequence
RNAT5	RNA thermometer/ iGEM Registry	TACTAGAGGCCGCGACAAGCGGTCCGGGCGCCCTA GGGGCCCGGCGGAGACGGGCGCCGGAGGTGTCCGA CGCCTGCTCGTCAAGTTCTTGCTCCTTGGAGGATTA CTAG
RNAT6	RNA thermometer/ iGEM Registry	ATTCAAGGGTAATCAATTCCTTCCACACATCAGGAG TTAACATTATG
P*J23119*	Promoter/iGEM Registry	TTGACAGCTAGCTCAGTCCTAGGTATAATGCTAGC

Note: This table is the gene element sequences achieved from the iGEM Registry.

Supplementary Table S4. Information of cold-resistant genetic elements.

Gene	Protein	Source	Locus tag
deaD	ATP-dependent RNA helicase DeaD	*N. pentaromativorans* US6-1	NSU_4286
nusA	Transcription termination/antitermination protein NusA	*N. pentaromativorans* US6-1	NSU_2765
rnr	Ribonuclease R	*N. pentaromativorans* US6-1	NSU_4344
degP	Periplasmic serine endoprotease DegP	*N. pentaromativorans* US6-1	NSU_1292
hfq	RNA-binding protein Hfq	*N. pentaromativorans* US6-1	NSU_4320
srmB^{33A}	ATP-dependent RNA helicase SrmB	*Glaciecola* sp. 33A	CXF81_10810
hfq^{33A}	RNA-binding protein Hfq	*Glaciecola* sp. 33A	CXF81_04330
deaD^{33A}	ATP-dependent RNA helicase DeaD	*Glaciecola* sp. 33A	CXF81_13210

Note: All information in this table is sourced from NCBI.

Supplementary Table S5. Information of cold-induced switches.

Name	Anti-RC	loop region	RC	Tm (℃)	ΔG (kcal/mol)
coldRNAT1	GUUGAAU GAGUUCG GUUAAGA	AAUAA	UCUUCCG CUCUUCC	29.5	-6.6
coldRNAT2	GUUGAAU GAGUUCG GUUAAGA	AAAAUAUA AA	UCUUCCG CUCUUCC	29.5	-5.7
coldRNAT3	GUUGAAU GAGUUCG GUUAAGA	AAAAUAUA UAUAAAA	UCUUCCG CUCUUCC	29.5	-5.3

Note: RNase E-recognized cleavage site (RC, UCUUCC) was marked with underline, and Tm value and G value are calculated by the UNAFold Web Server (http://www.unafold.org/).

Supplementary Table S6. Strains and plasmids used in this study.

Strain or plasmid	Description	Source
E. coli WM3064	Donor strain for conjugation	W. Metcalf
N. pentaromativorans US6-1	Wild type	Lab stock
pHGE-P*tac*	Km^r, IPTG-inducible P*tac* expression vector	Lab stock
pHGE-P*tac-grpE*	Overexpression plasmid containing *grpE* gene fragment	This study
pHGE-P*tac-danK*	Overexpression plasmid containing *danK* gene fragment	This study
pHGE-P*tac-dnaJ*	Overexpression plasmid containing *dnaJ* gene fragment	This study
pHGE-P*tac-groES*	Overexpression plasmid containing *groES*gene fragment	This study
pHGE-P*tac-groEL*	Overexpression plasmid containing *groEL* gene fragment	This study
pHGE-P*tac*-$grpE^{AA2-29}$	Overexpression plasmid containing $grpE^{AA2-29}$ gene fragment	This study
pHGE-P*tac-deaD*	Overexpression plasmid containing *deaD* gene fragment	This study
pHGE-P*tac-rnr*	Overexpression plasmid containing *rnr* gene fragment	This study
pHGE-P*tac-nusA*	Overexpression plasmid containing *nusA* gene fragment	This study
pHGE-P*tac-otsB*	Overexpression plasmid containing *otsB* gene fragment	This study
pHGE-P*tac-degP*	Overexpression plasmid containing *degP* gene fragment	This study
pHGE-P*tac-hfq*	Overexpression plasmid containing *hfq* gene fragment	This study
pHGE-P*tac*-$srmB^{33A}$	Overexpression plasmid containing $srmB^{33A}$ gene fragment	This study
pHGE-P*tac*-$deaD^{33A}$	Overexpression plasmid containing $deaD^{33A}$ gene fragment	This study
pHGE-P*tac*-hfq^{33A}	Overexpression plasmid containing hfq^{33A} gene fragment	This study

Supplementary Table S6. Strains and plasmids used in this study (continued).

Strain or plasmid	Description	Source
pHGE-P*tac*-*srmB*^{33A}	Overexpression plasmid containing *srmB*^{33A} controlled by P*tac*	This study
pHGE-P*tac*-*deaD*^{33A}	Overexpression plasmid containing *deaD*^{33A} controlled by P*tac*	This study
pHGE-P*tac*-*hfq*^{33A}	Overexpression plasmid containing *hfq*^{33A} controlled by P*tac*	This study
pHGE-P*J23119*-RNAT1-*sfgfp*	Heat-induced expression plasmid containing RNAT1 controlling *sfgfp*	This study
pHGE-P*J23119*-RNAT2-*sfgfp*	Heat-induced expression plasmid containing RNAT2 controlling *sfgfp*	This study
pHGE-P*J23119*-RNAT3-*sfgfp*	Heat-induced expression plasmid containing RNAT3 controlling *sfgfp*	This study
pHGE-P*J23119*-RNAT4-*sfgfp*	Heat-induced expression plasmid containing RNAT4 controlling *sfgfp*	This study
pHGE-P*J23119*-RNAT5-*sfgfp*	Heat-induced expression plasmid containing RNAT5 controlling *sfgfp*	This study
pHGE-P*J23119*-RNAT6-*sfgfp*	Heat-induced expression plasmid containing RNAT6 controlling *sfgfp*	This study
pHGE-P*J23119*-RC-*sfgfp*	Expression plasmid containing RC	This study
pHGE-P*J23119*-coldRNAT1-*sfgfp*	Cold-induced expression plasmid containing coldRNAT1 controlling *sfgfp*	This study
pHGE-P*J23119*-coldRNAT2-*sfgfp*	Cold-induced expression plasmid containing coldRNAT2 controlling *sfgfp*	This study
pHGE-P*J23119*-coldRNAT3-*sfgfp*	Cold-induced expression plasmid containing coldRNAT3 controlling *sfgfp*	This study
pHGE-P*J23119*-RNAT5-*grpE*$^{AA2-29}$	Expression plasmid containing *grpE*$^{AA2-29}$ gene fragment and heat-induced switch RNAT5 controlling *sfgfp*	This study
pHGE-P*J23119*-coldRNAT2-*srmB*^{33A}	Expression plasmid containing *srmB*^{33A} gene fragment and cold-induced switch coldRNAT2	This study
pHGE-P*J23119*-RNAT5-*grpE*$^{AA2-29}$ -P*J23119*-cold RNAT2-*srmB*^{33A}	Expression plasmid containing *grpE*$^{AA2-29}$ gene fragment, and heat-induced switch RNAT5, *srmB*^{33A} gene fragment, and cold-induced switch coldRNAT2	This study

Supplementary Table S7. Sequence of the primers used in this study.

Primer	Nucleotide sequence (5' to 3')
grpE-F	TTCACACAGGAGAGAATTCAATGACTGAAGACAAGACTCA
grpE-R	GCTTGGATCCTCGAGCTCCATCAGTCGGGCTTCTTGGCCA
dnaK-F	TTCACACAGGAGAGAATTCAATGGGAAAAGTAATTGGTAT
dnaK-R	GCTTGGATCCTCGAGCTCCATCAGCCCTTGTTTTCGTCGA
dnaJ-F	TTCACACAGGAGAGAATTCAGTGTCAGCTACCGAAATAGA
dnaJ-R	GCTTGGATCCTCGAGCTCCACTATTCGGTCAGGTCGCTCC
groEL-F	TTCACACAGGAGAGAATTCAATGACTTTCCGCCCGCTGCA
groEL-R	GCTTGGATCCTCGAGCTCCACAGGCGATGACGCCGAGAA
groES-F	TTCACACAGGAGAGAATTCAATGCATTTCCGACCTTTGCA
groES-R	GCTTGGATCCTCGAGCTCCATACGCCGCCTTCTTGAGTTC
deaD-F	GAGAGAATTCATGGAGCTCGAATGACATTTACCCGGGTTC
deaD-R	AAACAGCCAAGCTTGGATCCTCAGTCTCCCCGGCGATCCT
rnr-F	GAGAGAATTCATGGAGCTCGAATGAACCGCTTGAAAGCCA
rnr-R	AAACAGCCAAGCTTGGATCCCTAGCGCTTCTTGCGTCCCT
nusA-R	AAACAGCCAAGCTTGGATCCTCATTGCGAGGATTCCGCAT
degP-F	TTCACACAGGAGAGAATTCAATGGTGTCGAACACCGTCGA
degP-R	GCTTGGATCCTCGAGCTCCATCAGCGGATGCGGACGGGCA
hfq-F	TTCACACAGGAGAGAATTCAGTGAGCGCCGGAAATTCGGC
hfq-R	GCTTGGATCCTCGAGCTCCATCAATCTTCGGCTTCTTCTT
33A-*srmB*-F	TTCACACAGGAGAGAATTCAATGTTTGATCAATTAGATTT
33A-*srmB*-R	AAACAGCCAAGCTTGGATCCCTACGCCTCTTTTGATTTAG
33A-*hfq*-F	TTCACACAGGAGAGAATTCAATGGCTAAAGGTCAATCACT
33A-*hfq*-R	AAACAGCCAAGCTTGGATCCTTATTCGCCTTCACCGTCAC
33A-*deaD*-F	TTCACACAGGAGAGAATTCAATGACCGACACTGTCGATGT
33A-*deaD*-R	AAACAGCCAAGCTTGGATCCCTAACTTTTACGTTTTGGCG
29-*grpE*-F	TTCACACAGGAGAGAATTCAATGGAGGAGCGGAACCACGA
29-*grpE*-R	GCTTGGATCCTCGAGCTCCACTACTCCAGGTCCGCCTCTT
RNAT1-F	AAAAATATATATAAAAAGGAGAGAATTCAATG
RNAT1-R	ATATTTTTAGCGGAGAGAGGTTCACCGTGGTGTGAAATTG
RNAT2-F	AAAAATATATATAAAAAGGAGAGAATTCAATG
RNAT2-R	ATATTTTTACGAGAGAATTGAATGGTGTGAAATTG
RNAT3-F	AAAAATATATATAAAAAGGAGAGAATTCAATG
RNAT3-R	ATATTTTTACGAGAGAATTGTAATGGTGTGAAATTG

Supplementary Table S7. Sequence of the primers used in this study (continued).

Primer	Nucleotide sequence (5' to 3')
RNAT4-F	AAAAATATATATAAAAAGGAGAGAATTCAATG
RNAT4-R	ATATTTTTAGGACCCACGAATTCAATGGTGTGAAATTG
RNAT5-F	AATCATCGGCTCGTATAATGTACTAGAGGCCGCGACAAGC
RNAT5-R	TCGTGGTTCCGCTCCTCCATCTAGTAATCCTCCAAGGAGC
RNAT6-F	AATTCCTTCCACACATCAGGAGTTAACATTATGCGTAAAGGCGAAGAGCT
RNAT6-R	GTGTGGAAGGAATTGATTACCCTTGAATCTCTAGTACATTATACGAGCCG
RC-F	AAAATCTTCCGCTCTTCCTGTGGAATTGTGAGCGGA
RC-R	GGAAGATTTTAATTTTTTCATTATACGAGCCGATGA
coldRNAT1-F	TTAAAATCTTCCGCTCTTCCGGAATTGTGAGCGGATAACA
coldRNAT1-R	GCGGAAGATTTTAATTTTTTACACATTATACGAGCCGATG
coldRNAT2-F	TTAAGAAAAATATAAATCTTCCGCTCTTCCGGAATTGTGAGCGGATAACA
coldRNAT2-R	TTATATTTTTCTTAACCGAACTCATTCAACACACATTATACGAGCCGATG
coldRNAT3-F	AAAATATAAATCTTCCGCTCTTCCG
coldRNAT3-R	TTTATATTTTTCTTAACCGAACTCA
RTAS-F	ATTCACCACACCGGTAAACC
RTAS-R	ACCGGTGCGTTCACCGACAA
TAS-F	GGTTTACCGGTGTGGTGAATACCGGAGCTGTTGACAATTA
TAS-R	TTGTCGGTGAACGCACCGGTATGCCTGGCAGTTCCCTACT
P*J23119*-F	GCTCAGTCCTAGGTATAATGCTAGCTACTAGAGGCCGCGACAAGC
P*J23119*-R	TACCTAGGACTGAGCTAGCTGTCAATTATTGACTACCGGAAGCAG
coldRNAT2-F	TTAAGAAAAATATAAATCTTCCGCTCTTCCGGAATTGTGAGCGGATAACA
coldRNAT2-R	TTATATTTTTCTTAACCGAACTCATTCAACACACATTATACGAGCCGATG
RNAT5-F	AATCATCGGCTCGTATAATGTACTAGAGGCCGCGACAAGC
RNAT5-R	TCGTGGTTCCGCTCCTCCATCTAGTAATCCTCCAAGGAGC
